# The role of glycaemic and lipid risk factors in mediating the effect of BMI on coronary heart disease: a two-step, two-sample Mendelian randomisation study

**DOI:** 10.1007/s00125-017-4396-y

**Published:** 2017-09-09

**Authors:** Lin Xu, Maria Carolina Borges, Gibran Hemani, Debbie A. Lawlor

**Affiliations:** 1 School of Public Health, Sun Yat-sen University, Guangzhou, 510080 Guangdong People’s Republic of China; 2 MRC Integrative Epidemiology Unit, University of Bristol, Rm OS11, Oakfield House, Oakfield Grove, Bristol, BS8 2BN UK; 3 School of Public Health, University of Hong Kong, Hong Kong Special Administrative Region, People’s Republic of China; 4 Postgraduate Program in Epidemiology, Federal University of Pelotas, Pelotas, Brazil; 5 School of Social and Community Medicine, University of Bristol, Bristol, UK

**Keywords:** Body mass index, Cardiovascular disease risk factors, Coronary heart disease, Mediation, Mendelian randomisation

## Abstract

**Aims/hypothesis:**

The extent to which effects of BMI on CHD are mediated by glycaemic and lipid risk factors is unclear. In this study we examined the effects of these traits using genetic evidence.

**Methods:**

We used two-sample Mendelian randomisation to determine: (1) the causal effect of BMI on CHD (60,801 case vs 123,504 control participants), type 2 diabetes (34,840 case vs 114,981 control participants), fasting glucose (*n* = 46,186), insulin (*n* = 38,238), HbA_1c_ (*n* = 46,368) and LDL-cholesterol, HDL-cholesterol and triacylglycerols (*n* = 188,577); (2) the causal effects of glycaemic and lipids traits on CHD; and (3) the extent to which these traits mediate any effect of BMI on CHD.

**Results:**

One SD higher BMI (~ 4.5 kg/m^2^) was associated with higher risk of CHD (OR 1.45 [95% CI 1.27, 1.66]) and type 2 diabetes (1.96 [95% CI 1.35, 2.83]), higher levels of fasting glucose (0.07 mmol/l [95% CI 0.03, 0.11]), HbA_1c_ (0.05% [95% CI 0.01, 0.08]), fasting insulin (0.18 log pmol/l [95% CI 0.14, 0.22]) and triacylglycerols (0.20 SD [95% CI 0.14, 0.26]) and lower levels of HDL-cholesterol (−0.23 SD [95% CI −0.32, −0.15]). There was no evidence for a causal relation between BMI and LDL-cholesterol. The causal associations of higher triacylglycerols, HbA_1c_ and diabetes risk with CHD risk remained after performing sensitivity analyses that considered different models of horizontal pleiotropy. The BMI–CHD effect reduced from 1.45 to 1.16 (95% CI 0.99, 1.36) and to 1.36 (95% CI 1.19, 1.57) with genetic adjustment for triacylglycerols or HbA_1c_, respectively, and to 1.09 (95% CI 0.94, 1.27) with adjustment for both.

**Conclusions/interpretation:**

Increased triacylglycerol levels and poor glycaemic control appear to mediate much of the effect of BMI on CHD.

**Electronic supplementary material:**

The online version of this article (doi:10.1007/s00125-017-4396-y) contains peer-reviewed but unedited supplementary material, which is available to authorised users.

## Introduction

Greater BMI is a risk factor for a wide range of adverse health outcomes, including CHD the leading cause of death worldwide. Whilst preventing overweight and obesity is an important public health aim, the substantial and increasing number of people with a high BMI highlights the need for secondary prevention that aims to reduce risk of the main disease outcomes of high BMI, such as CHD, by targeting causal mediators. This is also important because beyond bariatric surgery there are no effective and sustainable treatments for those who are obese [1].

Large prospective population studies show that higher BMI is associated with adverse blood lipid levels, higher fasting glucose and insulin, type 2 diabetes mellitus and CHD. RCTs show that elevated triacylglycerols, LDL-cholesterol, glucose and BP increase the risk of CHD [2, 3]. Thus, the association of BMI with CHD could be mediated by these established modifiable risk factors. However, the common method used to test for mediation, by observing how much the confounder-adjusted multivariable association between a risk factor (e.g. BMI) and outcome (e.g. CHD) reduces with further adjustment for potential mediators [4], has been shown to be biased in many situations [5].

**Fig. 1:**
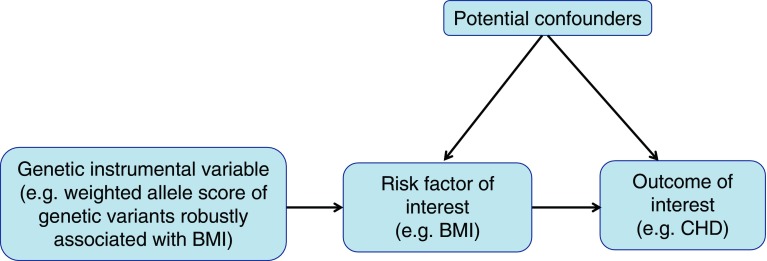
Summary of MR and its assumptions. The underlying assumptions of MR are that: (1) the genetic instrumental variable(s) are robustly related to the risk factor of interest (here BMI; this is illustrated by the arrow from the genetic instruments to BMI); (2) there is no relationship between any confounders of the risk factor (BMI) and outcome (CHD) and the genetic instrumental variable (illustrated by the lack of any arrow between these confounders and the genetic instrument); and (3) there is no path from the genetic instrument to the outcome other than through its relationship to the risk factor (illustrated by the lack of any arrow that goes directly from the genetic instrument to the outcome). Empirical evidence suggests that the most likely of these three assumptions to be violated, and result in potentially biased results, is the last one. This may be violated in MR studies by horizontal pleiotropy (i.e. where the genetic instrument[s] affect other factors which, independent of their impact on the risk factor of interest, influence the outcome). If this horizontal pleiotropy is present then the MR estimate of the effect of a risk factor on outcome will be biased, it will actually be the combined effect of that risk factor and any other (pleiotropic) paths from the genetic instruments to outcome. The bias could be an exaggeration of the true effect (if the horizontal pleiotropic paths are in the same direction as that of the main risk factor of interest) or a diminution of the true effect (if the horizontal pleiotropic effect is in the opposite direction of the risk factor of interest). There are a number of different statistical methods that can be used to estimate causal MR effects. Many of these are related to the ratio, which is intuitive. If the assumptions above are correct then the causal effect of the risk factor (BMI) on outcome (CHD) is the ratio of ‘the association of genetic instruments with CHD’ to ‘the association of genetic instrument with BMI’. Valid MR estimates can be obtained using two (independent) samples for the association of the genetic instrument with outcome and the association of genetic instrument with risk factor [16]. There are some advantages of this two-sample MR approach over the one-sample approach (where both parts of the ratio are obtained from the same sample), including the potential to gain very large sample sizes by using publicly available aggregate genome-wide data as we have done here and apply novel methods for testing horizontal pleiotropy that have been developed for use in two-sample MR with aggregate GWAS data (see the Methods section and the ESM for detailed descriptions of these)

Mendelian randomisation (MR), the use of genetic variants as instrumental variables to test the causal effect of risk factors on outcomes, is unlikely to be biased by the extensive confounders of multivariable observational analyses, is less prone to measurement error [6] and, because genetic variants are fixed at conception, cannot be biased by reverse causality [7, 8]. As such, MR has been used increasingly over the past decade to provide more robust estimates for the causal effect of many risk factors on a range of health outcomes, with results from MR closely resembling those from RCTs where both are available (e.g. the effect of LDL-cholesterol [9] and systolic BP [10] on CHD). Recently, methods have been developed for its use in testing causal mediation using a two-step approach that is considerably less prone to the biases inherent in the common multivariable approach [5]. Figure 1 provides a brief description of MR and its assumptions.

Previous MR studies using data from three collections have shown that higher BMI causally relates to higher risk for CHD (the results of our meta-analysis of these previous MR studies are presented in the electronic supplementary material [ESM] Fig. 1) [11–14]. These studies used one-sample MR and were unable to undertake sensitivity analyses that have been developed for testing likely bias by pleiotropy [15]. The number of cases of CHD varied from 3062 to 11,056, which are modest for MR studies. Although MR is likely to be less biased than conventional multivariable approaches, it usually requires a considerably larger sample size. Only one of these MR studies analysed potential mediators of the impact of BMI on CHD. It concluded that LDL-cholesterol, remnant cholesterol and systolic BP, explained 8%, 7% and 7%, respectively, of the effect of BMI on CHD [14]. That study was unable to explore potential mediation by insulin sensitivity or hyperglycaemia, which are strongly influenced by BMI and are strong risk factors for CHD. Here, we aimed to investigate the mediating effects of lipid and insulin/glycaemic traits on the effect of BMI on CHD using a large MR study, including over 60,000 individuals with CHD, and to analyse a wider set of potential mediators including glycaemic traits (fasting glucose and insulin, HbA_1c_, type 2 diabetes) than previous studies.

## Methods

We used two-step two-sample MR [5, 16] with publicly available datasets that provide genome-wide association results for BMI, glycaemic traits, lipids and CHD. Two-sample MR refers to the use of different datasets (samples) to obtain the gene–risk factor (e.g. BMI) and gene–outcome (e.g. CHD) associations. First, we tested the effects of BMI on CHD, and then the effects of potential mediation using two-step MR. In step one we tested causal effects of BMI on potential mediators and in step two the causal effects of potential mediators on CHD [5].

### Data sources

#### Genetic instrumental variable for BMI

From the most updated genome-wide associations studies (GWAS) on BMI, the Genetic Investigation of ANthropometric Traits (GIANT) consortium, we obtained 77 SNPs, identified from the primary meta-analysis of 322,154 European-descent individuals, independently contributing to BMI at genome-wide significance (*p* < 5 × 10^−8^) [17]. These variants were defined as being independent of each other on the basis of low correlation (*R* ^2^ < 0.1) in HapMap22 or the 1000 Genome project data. These 77 SNPs account for 2.4% of BMI phenotypic variance [17]. For sensitivity analyses, we included 20 SNPs from the secondary analysis of this GWAS [17]; these include some SNPs that did not reach genome-wide significance in Europeans.

#### Potential mediators

Associations of SNPs with the phenotypes were extracted from publicly available GWAS consortia. Data on type 2 diabetes mellitus GWAS correlates was obtained from the DIAbetes Genetics Replication And Meta-analysis (DIAGRAM, http://diagram-consortium.org/downloads.html, accessed on 22 June 2016), which includes 34,840 case and 114,981 control participants of European origin [18]. Genetic associations with fasting insulin (*n* = 38,238), fasting glucose (*n* = 46,186) and HbA_1c_ (*n* = 46,368) were obtained from the Meta-Analyses of Glucose and Insulin-related traits Consortium (MAGIC, http://www.magicinvestigators.org/, accessed on 22 June 2016); the participants were of European ancestry without diabetes [19]. Genetic associations with HDL-cholesterol, LDL-cholesterol and triacylglycerols in 188,577 Europeans were obtained from the Global Lipids Genetics Consortium (GLGC) investigators (http://csg.sph.umich.edu/abecasis/public/lipids2013/, accessed on 22 June 2016) [20].

#### Study outcome: CHD

Data on coronary artery disease/myocardial infarction were obtained from the Coronary ARtery DIsease Genome wide Replication And Meta-analysis (CARDIoGRAM) plusC4D investigators (www.CARDIOGRAMPLUSC4D.ORG, accessed on 22 June 2016) [21]. This includes 60,801 CHD case and 123,504 control participants. We first searched the CARDIoGRAMplusC4D 1000 Genomes-based GWAS, a meta-analysis of GWAS studies of mainly European, South Asian and East Asian descent imputed using the 1000 Genomes phase 1 v3 training set with 38 million variants [22]. If no summary data on the gene–CHD association were found from the 1000 Genomes data, then we screened in CARDIoGRAMplusC4D Metabochip. If the targeted SNPs were not found in either the 1000 Genomes or the CARDIoGRAMplusC4D Metabochip, we then screened CARDIoGRAM GWAS.

The genetic variants used as instrumental variables for CHD, BMI and CHD risk factors (potential mediators) are all shown in ESM Tables 1–9.

### Statistical analysis

As an indication of the strength of the association between genetic instruments and phenotypes, we report the proportion of variation in BMI and all mediators explained by their genetic variant instruments and also the F-statistic for the regression of BMI and all mediators on their genetic instruments. The proportion of the BMI–CHD effect that is explained by a group of mediators will be estimated with bias if the mediators are related to each other, and/or if the outcome has an effect on the mediator (i.e. there is reverse causality) and the instrument affects the mediators through the outcome. Therefore, we tested for potential bi-directional causal effects of BMI, potential mediators and CHD with each other using the inverse variance weighted (IVW) approach described below.

Horizontal pleiotropy, where the genetic variant influences the outcome through a pathway other than the exposure, violates an assumption of MR and can bias causal estimates. To guard against this we used three different analytical approaches for both step one (effect of BMI on CHD and potential mediators) and step two (effect of potential mediators on CHD) of the two-step MR mediation approach. Each of the three methods assumes different models of horizontal pleiotropy. The value of comparing results from all three is that we have more confidence in results that are consistent across the different methods. Full details of these approaches, including their different assumptions, are provided in Table 1 and ESM Methods 1.

**Table 1:** Summary of the three methods used for MR analysis

	IVW	Weighted-median	MR-Egger
Assumption	All genetic instrumental variables are valid or any horizontal pleiotropic effects of instruments are balanced	No more than 50% of the weight of the estimate is from invalid genetic instrumental variables No single instrumental variable contributes >50% of the weight	InSIDE (instrument strength independent of direct effect) assumption, which states that the effect of the instrument on the exposure is not correlated with any direct effect of the instrument on the outcome
Equation	${\widehat{\upbeta}}_{IVW}=\frac{\sum_{K=1}^K{E}_k{D}_k{\upsigma}_{Dk}^{-2}}{\sum_{K=1}^K{E}_k^2{\upsigma}_{Dk}^{-2}}$ ${SE}_{{\widehat{\upbeta}}_{IVW}}=\sqrt{\frac{1}{\sum_{k=1}^k{E}_K^2{\upsigma}_{Dk}^{-2}}}$ *E*_*K*_ is the mean change in exposure level per additional effect allele of SNP k; *D*_*k*_ is the mean change in disease outcomes (e.g. log odds of CHD or levels of other cardiovascular disease risk factors) per additional effect allele of SNP k with SE σ_*Dk*_	Weighted-median estimator is the median of a distribution having estimate β_j_ as: P_j_ = 100(*S*_*j*_ − *W*_*j*_/2)^th^ percentile P is the percentile for the j^th^ ordered ratio estimate; *W*_*j*_ is the weight given to the j^th^ ordered ratio estimate, proportional to the inverse of the instrumental variable variance, and *S*_*j*_ is the sum of weights up to and including the weight of the j^th^ ordered ratio estimates, calculated using the following equation: $Sj={\sum}_{K=1}^j{W}_k$	MR-Egger uses a weighted linear regression of the gene–outcome coefficients θ_j_ on the gene–exposure coefficients δ_j_: θ_j_ = β_0E_ + β_E_ × δ_j_ All the δ_j_ associations are orientated to be positive. If β_0E_ is truly zero (or were constrained to be zero) the MR-Egger slope estimate β_E_ is the same as the β from IVW
Application	The IVW estimate is a statistically efficient method but it can be biased even if just one genetic variant is invalid (i.e. if just one variant has horizontal pleiotropic effects)	The weighted-median estimator is a modification of the simple median approach and takes account of the variance of the individual genetic instruments	The MR-Egger method is used to test for directional horizontal pleiotropy and correct for this in MR analyses

**Fig. 2:**
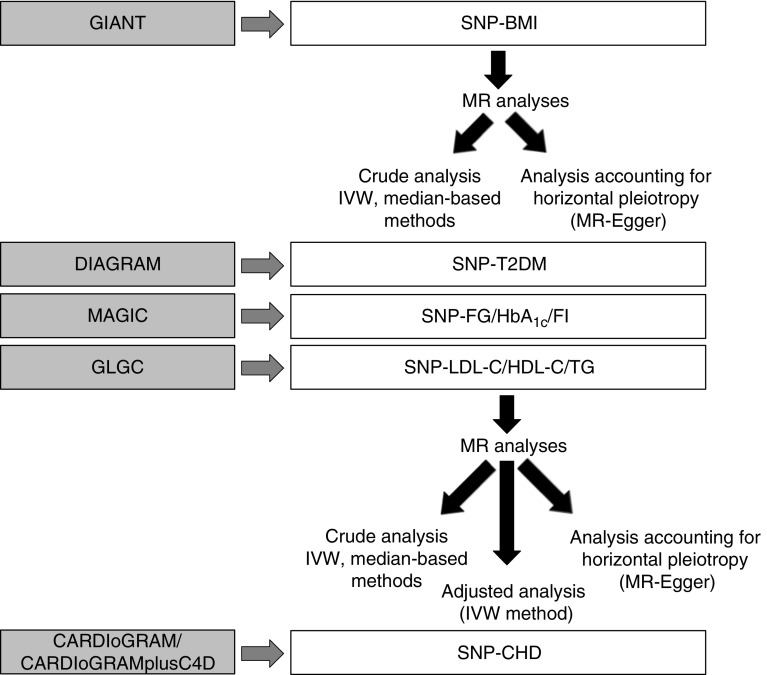
Analysis diagram. Summary data for SNP phenotypes were extracted from GWAS consortia datasets (GIANT, CARDIoGRAM, C4D, DIAGRAM, MAGIC and GLGC). MR estimates of BMI on mediators (type 2 diabetes [T2DM], fasting glucose [FG], fasting insulin [FI], HbA_1c_, LDL-cholesterol [LDL-C], HDL-cholesterol [HDL-C] and triacylglycerols [TG]), and of BMI and mediators on CHD were derived using the IVW method

To estimate the effect of BMI on CHD taking account of genetically determined potential mediators, we used the IVW MR method, adjusting for the SNP–potential mediator effect [23]. The proportion of the effect that is mediated by any of the potential mediators was estimated by the changes in the total effect of the genetically determined BMI on CHD risk (for more details see ESM Methods 2). This method assumes that mediators are continuously measured variables and as type 2 diabetes is dichotomised we did not assess the proportion of the BMI–CHD effect due to type 2 diabetes. An analysis diagram is shown in Fig. 2. All statistical analysis was performed using STATA 13.1 (Stata Corp LP, College Station, TX, USA) and R (version 3.2.5, the R Foundation for statistical Computing, Vienna, Austria) software.

## Results

The proportion of variation explained by all of the variants that we used as instrumental variables for the potential mediators varied from 1.2% (for fasting insulin) to 5.7% (for type 2 diabetes) (ESM Tables 1–8). The first stage F-statistic for all of the MR analyses (i.e. for the regression of BMI and each of the mediators on their genetic variant instrument variables) were very large (> 500).

### Relationships between potential mediators and CHD

As expected, we observed evidence for association between fasting plasma glucose and type 2 diabetes, and that both fasting plasma glucose and type 2 diabetes were associated with HbA_1c_ (Table 2). LDL-cholesterol, HDL-cholesterol and triacylglycerols were associated with each other. CHD appears to be causally positively related to type 2 diabetes, but was not related to other potential mediators (Table 2).

**Table 2:** MR estimates^a^ of risk factors on each other and on CHD and type 2 diabetes

Outcome	Exposure								
	BMI, SD	T2DM	FPG, mmol/l	HbA_1c_, %	FI, log-pmol/l	LDL-C, SD	HDL-C, SD	TG, SD	CHD
BMI, SD	0	−0.07 (0.009)***	0.007 (0.04)	0.04 (0.05)	−0.51 (0.14)***	−0.04 (0.01)*	0.0008 (0.01)	−0.004 (0.02)	−0.015 (0.01)
T2DM	0.67 (0.19)***	0	1.23 (0.14)***	0.36 (0.2)	0.53 (0.45)	−0.09 (0.06)	−0.03 (0.05)	0.05 (0.08)	0.10 (0.05)*
FPG, mmol/l	0.07 (0.02)***	0.08 (0.007)***	0	0.07 (0.07)	0.05 (0.07)	−0.04 (0.01)**	−0.002 (0.01)	−0.01 (0.02)	0.01 (0.01)
HbA_1c_, %	0.05 (0.03)***	0.03 (0.01)**	0.45 (0.04)***	0	−0.09 (0.1)	−0.01 (0.02)	−0.02 (0.02)	−0.03 (0.02)	0.01 (0.02)
HbA_1c_, mmol/mol	0.55 (0.33)***	0.33 (0.11)**	4.92 (0.44)***	0	−0.98 (1.09)	−0.11 (0.22)	−0.22 (0.22)	−0.33 (0.22)	0.11 (0.22)
FI, log-pmol/l	0.18 (0.03)***	0.06 (0.009)***	0.05 (0.05)	−0.06 (0.06)	0	−0.03 (0.02)	0.007 (0.02)	0.02 (0.02)	−0.008 (0.01)
LDL-C, SD	−0.05 (0.07)	0.02 (0.01)	0.02 (0.04)	0.09 (0.05)	0.27 (0.14)*	0	−0.21 (0.02)***	0.19 (0.03)***	−0.03 (0.02)
HDL-C, SD	−0.23 (0.04)***	−0.003 (0.01)	0.04 (0.04)	0.11 (0.05)*	0.52 (0.16)***	−0.18 (0.02)***	0	−0.47 (0.03)***	−0.02 (0.02)
TG, SD	0.20 (0.03)***	0.02 (0.01)	0.03 (0.03)	−0.02 (0.05)	−0.21 (0.16)	0.07 (0.03)*	−0.16 (0.01)***	0	0.001 (0.02)
CHD	0.37 (0.07)***	0.10 (0.03)***	0.19 (0.09)*	0.31 (0.12)*	−0.49 (0.31)	0.49 (0.05)***	−0.13 (0.04)**	0.21 (0.05)***	0

^a^All results are β coefficients (SE) from the MR instrumental variable estimates using IVW and so reflect differences in mean outcome per one unit difference of the exposures for continuously measured outcomes and difference in log odds for binary outcomes (CHD/type 2 diabetes)

*p < 0.05, **p < 0.01, ***p < 0.001

FI, fasting insulin; FPG, fasting plasma glucose; HDL-C, HDL-cholesterol; LDL-C, LDL-cholesterol; T2DM, type 2 diabetes mellitus; TG, triacylglycerols

### Effects of BMI on CHD and glycaemic and lipid traits

There was consistent support across all three MR methods for a causal effect of higher BMI on higher CHD and type 2 diabetes risk, and higher levels of fasting glucose, HbA_1c_, fasting insulin and triacylglycerols, together with lower HDL-cholesterol (Table 3). None of the methods supported a causal effect of BMI on LDL-cholesterol (Table 3 and ESM Table 10).

**Table 3:** MR estimates of BMI (SD, 1 SD = 4.5 kg/m^2^) on cardiovascular risk factors and CHD

Exposure: BMI (*n* = 322,154)	Effect estimate	95% CI	*p* value
CHD (*n* = 60,801 case and 123,504 control participants)^a^			
IVW	1.45	1.27, 1.66	< 0.001
Weighted-median	1.44	1.24, 1.67	< 0.001
MR-Egger regression			
Slope	1.55	1.26, 1.91	< 0.001
Intercept (directional pleiotropy)	1.00	0.99, 1.00	0.50
Type 2 diabetes mellitus (*n* = 34,840 case and 114,981 control participants)^a^			
IVW	1.96	1.35, 2.83	< 0.001
Weighted-median	2.63	2.16, 3.21	< 0.001
MR-Egger regression			
Slope	3.42	2.63, 4.46	< 0.001
Intercept (directional pleiotropy)	0.98	0.98, 0.99	< 0.001
Fasting glucose, mmol/l (*n* = 46,186)^b^			
IVW	0.07	0.03, 0.11	< 0.001
Weighted-median	0.08	0.05, 0.12	< 0.001
MR-Egger regression			
Slope	0.09	0.036, 0.15	< 0.001
Intercept	−0.0007	−0.002, 0.001	0.37
HbA_1c_, % (*n* = 46,368)^b^			
IVW	0.05	0.01, 0.08	0.005
Weighted-median	0.09	0.04, 0.14	< 0.001
MR-Egger regression			
Slope	0.09	0.008, 0.16	0.03
Intercept	−0.001	−0.003, 0.001	0.31
Fasting insulin, log-pmol/l (*n* = 38,238)^b^			
IVW	0.18	0.14, 0.22	< 0.001
Weighted-median	0.18	0.12, 0.24	< 0.001
MR-Egger regression			
Slope	0.16	0.07, 0.25	< 0.001
Intercept	0.0007	−0.002, 0.003	0.60
LDL-cholesterol, SD (1 SD = 1.0 mmol/l) (*n* = 188,577)^b^			
IVW	−0.05	−0.19, 0.09	0.50
Weighted-median	−0.01	−0.08, 0.05	0.66
MR-Egger regression			
Slope	−0.10	−0.184, − 0.02	0.02
Intercept	0.0016	−0.001, 0.004	0.19
HDL-cholesterol, SD (1 SD = 0.40 mmol/l) (*n* = 188,577)^b^			
IVW	−0.23	−0.32, −0.15	< 0.001
Weighted-median	−0.21	−0.27, −0.16	< 0.001
MR-Egger regression			
Slope	−0.23	−0.307, −0.15	< 0.001
Intercept	−0.0001	−0.002, 0.002	0.90
Triacylglycerol, SD (1 SD = 1.024 mmol/l) (*n* = 188,577)^b^			
IVW	0.20	0.14, 0.26	< 0.001
Weighted-median	0.21	0.15, 0.27	< 0.001
MR-Egger regression			
Slope	0.17	0.09, 0.24	< 0.001
Intercept	0.001	−0.001, 0.003	0.37

^a^Binary outcome—effect estimate is the OR for a 1 SD increase in BMI

^b^Continuously measured outcome—effect estimate is the difference in mean in the unit provided in column 1 for a 1 SD increase in BMI

### Effects of potential mediators on CHD

There was broadly consistent support across all three MR methods for a positive effect of type 2 diabetes, HbA_1c_, triacylglycerols and LDL-cholesterol on CHD risk (Table 4). For type 2 diabetes the MR-Egger 95% CI just included the null value, but this method has lower statistical power than the others and the point estimates were similar across all methods. For triacylglycerols the estimate of effect (slope) from MR-Egger was a little weaker than for all of the other methods (e.g. 1.24 vs 1.13 comparing the IVW and MR-Egger methods), suggesting that some but not all of the effect of triacylglycerols estimated by IVW and other methods might be due to horizontal pleiotropy. In IVW and the median method analyses, there is evidence for the causal association of lower HDL-cholesterol and higher fasting glucose and insulin with higher risk of CHD. However, for all of these MR-Egger suggested that effects were largely due to horizontal pleiotropy, with effect estimates markedly attenuated to the null and the intercepts all being non-zero.

**Table 4:** MR estimates of cardiovascular risk factors on CHD

Risk factor	OR	95% CI	*p* value
Type 2 diabetes mellitus			
IVW	1.12	1.06, 1.18	< 0.001
Weighted-median	1.11	1.05, 1.17	< 0.001
MR-Egger regression			
Slope	1.07	0.99, 1.15	0.10
Intercept	1.01	1.00, 1.01	0.17
Fasting glucose, mmol/l			
IVW	1.31	1.09, 1.58	< 0.001
Weighted-median	1.21	1.01, 1.44	0.03
MR-Egger regression			
Slope	1.08	0.87, 1.35	0.50
Intercept	1.01	1.00, 1.01	0.04
HbA_1c_, %			
IVW	1.30	1.08, 1.56	0.01
Weighted-median	1.36	1.07, 1.74	0.01
MR-Egger regression			
Slope	1.66	1.03, 2.68	0.04
Intercept	0.99	0.97, 1.01	0.27
Fasting insulin, log-pmol/l			
IVW	2.80	1.89, 4.16	< 0.001
Weighted-median	2.61	1.61, 4.23	< 0.001
MR-Egger regression			
Slope	0.49	0.09, 2.59	0.40
Intercept	1.03	1.00, 1.05	0.04
LDL-cholesterol, SD (1 SD = 1.0 mmol/l)			
IVW	1.58	1.43, 1.75	< 0.001
Weighted-median	1.63	1.48, 1.80	< 0.001
MR-Egger regression			
Slope	1.74	1.59, 1.90	< 0.001
Intercept	0.99	0.98, 0.99	0.01
HDL-cholesterol, SD (1 SD = 0.4 mmol/l)			
IVW	0.86	0.78, 0.95	< 0.001
Weighted-median	0.88	0.81, 0.95	< 0.001
MR-Egger regression			
Slope	1.03	0.95, 1.12	0.46
Intercept	0.99	0.98, 0.99	< 0.001
Triacylglycerol, SD (1 SD = 1.024 mmol/l)			
IVW	1.24	1.10, 1.41	< 0.001
Weighted-median	1.23	1.11, 1.36	< 0.001
MR-Egger regression			
Slope	1.13	1.03, 1.24	0.01
Intercept	1.01	1.003, 1.01	< 0.001

### Mediating effects of lipids and glycaemic traits on BMI–CHD effects

We explored those potential mediators that had causal support from MR for both an effect of BMI on them (step one) and of the mediators on CHD (step two): type 2 diabetes, HbA_1c_ and triacylglycerols (Table 5). Our results suggested that triacylglycerols were an important mediator, with either type 2 diabetes or HbA_1c_ further contributing to mediation of BMI on CHD. The BMI–CHD effect reduced from 1.45 (95% CI 1.27, 1.66) to 1.16 (95% CI 0.99, 1.36) and 1.36 (95% CI 1.19, 1.57) with adjustment for the estimated effects of triacylglycerols and HbA_1c_, respectively, and to 1.09 (95% CI 0.94, 1.27) with adjustment for both.

**Table 5:** Multivariate separate-sample MR analysis of the effect of BMI (per SD, 1 SD = 4.5 kg/m^2^) on CHD

	OR	95% CI	*p* value	Mediation effect (%)
MR-IVW regression, crude	1.45	1.27, 1.66	< 0.001	
Multivariate model				
(1) Adjusted for triacylglycerol	1.16	0.99, 1.36	0.06	22
(2) Adjusted for HbA_1c_	1.36	1.19, 1.57	0.001	4
(3) Adjusted for type 2 diabetes	1.35	1.17, 1.56	0.001	–
(4) Adjusted for triacylglycerol + HbA_1c_	1.09	0.94, 1.27	0.25	38
(5) Adjusted for triacylglycerol + type 2 diabetes	1.10	0.94, 1.29	0.22	–

## Discussion

This is the first paper to explore the extent to which glycaemic traits mediate a causal path between BMI and CHD, whilst using statistical approaches that account for horizontal pleiotropy. Consistent with previous studies [11–14], but using a larger sample size and more genetic variants, we show that higher BMI causes greater CHD risk. Our results also suggest that triacylglycerols, HbA_1c_ and type 2 diabetes play important roles in causally mediating the effect of BMI on CHD. In contrast, our results do not support causal effects for the observed association between BMI and LDL-cholesterol, or for the associations of HDL-cholesterol, fasting glucose or insulin with CHD. Secondary prevention, which aims to reduce obesity-related CHD by targeting causal mediators is important because of the large, and increasing, proportions of people globally who are overweight or obese and the lack, currently, of scalable effective treatments for obesity. Treating causal mediators of the effect of BMI on CHD could mitigate its effect, but biases in conventional epidemiological methods for testing mediation have limited our understanding of which CHD risk factors mediate BMI effects. Our findings provide strong support for undertaking RCTs in obese people to test the effect of triacylglycerol reduction and glycaemic control on CHD risk.

Several MR studies have previously examined the association of BMI with CHD and CHD risk factors [11, 12, 14, 24, 25]. Our results are broadly consistent with those, including our finding of no evidence for the causal association between BMI and LDL-cholesterol [12, 24, 26]. This is further supported by two RCTs of bariatric surgery which found that intensive weight loss did not lower LDL-cholesterol [27, 28]. Consistent with our results, previous MR studies have also shown positive causal effects of type 2 diabetes, HbA_1c_, LDL-cholesterol and triacylglycerols with CHD [29–32], but not for causal effects of fasting glucose or HDL-cholesterol with CHD once horizontal pleiotropy has been accounted for. The discrepancy between finding a causal effect of type 2 diabetes and HbA_1c_ on CHD, but not of fasting glucose, might suggest that non-fasting (postprandial) glucose levels, more so than fasting levels, are most relevant for CHD risk and/or that long-term hyperglycaemia (as assessed by elevated HbA_1c_ and likely to be identified as being above the threshold required to diagnose type 2 diabetes) are important.

To our knowledge only one previous study has tried to explore potential mediation of the BMI–CHD effect in an MR framework. That study included 11,056 individuals with CHD and 75,627 control participants from Copenhagen and used only three BMI-related SNPs. It concluded that the effect of BMI on increased CHD risk was partly mediated through elevated levels of LDL-cholesterol, non-fasting remnant cholesterol and systolic BP [14]. The evidence for a mediating role of remnant cholesterol is entirely consistent with our findings here for triacylglycerols, as remnant cholesterol is the cholesterol content of triacylglycerol-rich lipoproteins, particularly so in this previous study where remnant cholesterol was not directly measured but estimated from other lipids using a method that would produce an extremely high correlation between (measured) triacylglycerols and estimated remnant cholesterol; mediation by remnant cholesterol is thus consistent with mediation by triacylglycerols [14].

Our study, the previous (Copenhagen) study [13, 14], other MR studies [12, 24, 26] and RCTs of bariatric surgery [33, 34] have found no evidence for a causal effect of BMI on LDL-cholesterol, which suggests it is unlikely to be an important mediator of BMI on CHD. However, since the previous study (despite finding no MR evidence for a causal effect of BMI on LDL-cholesterol) concluded that LDL-cholesterol was a partial mediator [14], we examined that possibility in our data. As expected we found no strong support for a mediating effect of LDL-cholesterol between BMI and CHD (ESM Table 12). We were unable to explore any mediating effect of BP in our study. This is because our approach uses publicly available aggregate genome-wide results and the International Consortium for Blood Pressure (ICBP) provides information on SNPs and BP associations without specifying the risk (or effect) allele for each SNP; thus the effect of BMI on BP cannot be assessed using the two-sample MR instrumental variable analysis.

CHD is a major cause of morbidity and mortality and its prevalence is increasing worldwide, partly because of the increasing prevalence of obesity. Our results indicate the extent to which acting on risk factors, such as triacylglycerols, HbA_1c_ and type 2 diabetes, might counteract the detrimental effects of obesity on CHD. They highlight the potential importance of using interventions that lower triacylglycerols and/or HbA_1c_ and type 2 diabetes specifically in those with obesity [35, 36]. There is evidence, including from MR studies, that statins affect triacylglycerols and remnant cholesterol, as well as LDL-cholesterol [37]. Furthermore, a rare variant in *APOC3* with a marked effect on triacylglycerol levels provides a potential target for drug development aimed at reducing triacylglycerol levels, independent of any statin effects [38, 39]. Thus, targets for reducing triacylglycerols exist and testing the effect of these in obese populations would be feasible. Previous RCTs have shown that the oral hypoglycaemic metformin reduces cardiovascular risk factors [40–42] in non-diabetic at-risk populations, including those who are obese, but its effect on CHD risk has yet to be established. Our results suggest that it might be cardioprotective in populations with high BMI and supports the development of RCTs to test its effect on CHD in these people.

### Strengths and limitations

Two-sample MR exploits the fact that it is not necessary to obtain both gene–exposure association (ratio denominator) and gene–outcome association (ratio numerator) from the same sample of participants. There are some advantages to obtaining them from two different sets of participants. For example, ‘winners’ curse’ [16] can bias true causal effects towards observational results in one-sample MR but is less likely to generate false-positive findings in two-sample MR. In addition, the weak instrument bias, which biases effects towards the confounded multivariable regression result in one-sample MR, biases the effect towards the null in two-sample MR (with non-overlapping datasets). The main advantage of two-sample MR is the increased statistical power, particularly in relation to testing effects on binary disease outcomes (i.e. CHD or type 2 diabetes) because of the use of summary data from GWAS consortia [16].

Our study is extremely large and uses genetic variants to avoid some of the key limitations of traditional multivariable regression approaches to mediation. Horizontal pleiotropy is one of the major concerns in relation to limitations of MR studies. However, to explore the potential effects of this pleiotropy, we used different MR methods (IVW, median-based estimators and MR-Egger) that have different assumptions and we assessed the consistency across each of these estimators. The mediators that we took forward into MR-based mediation analyses (triacylglycerols, HbA_1c_ and type 2 diabetes) had consistent causal effects across these different methods for both steps (i.e. the effect of BMI on them and of them on CHD). In the mediation analyses, where we include both genetically predicted triacylglycerols and HbA_1c_, we are assuming that these factors are not causally related to each other. We tested for causal relationships between potential mediators prior to our main two-step MR analyses and these do not suggest any causal effects between triacylglycerols and HbA_lc_ or other glycaemic traits. However, MR studies cannot completely rule out a causal relationship between the two. Previous large prospective studies showed triacylglycerols predicted the development of type 2 diabetes [43, 44], if this association is casual, the estimated mediation effect by dysglycaemia and triacylglycerols could be inflated. Our results would be biased if the mediators we have tested caused variation in BMI (i.e. there was reverse causality from mediators to BMI). If this were the case, we would expect a bi-directional MR effect between BMI and the mediating risk factors. However, we found no evidence that triacylglycerols or HbA_1c_ caused variation in BMI (though the causal effect of BMI to these mediators was present).

Whilst all three MR methods suggest a casual effect of triacylglycerols on CHD, the MR-Egger intercept suggests that directional horizontal pleiotropy may be exhibited by the instruments. It is plausible that the genetic variants we used as instruments for triacylglycerols also affect other remnant cholesterols or other lipids and those also contribute to mediating BMI effects on CHD. Another potential limitation to our study is that we have assumed no interaction between BMI and mediators, but we are not able to test for this because we have used aggregated genome-wide data. Previous observational studies suggest that the association between BMI and CHD may be modified by hypertension [45], but have not found effect modification by the glycaemic and lipid traits that we have examined here [46]. In two-sample MR, with independent samples, weak instrument bias can result in bias towards the null. In mediation analyses this could result in an underestimation of mediating effects. However, given our large sample size and the fact that our genetic instruments explained 2.1% and 2.4% of the variation in triacylglycerols and HbA_1c_, respectively, and had very large first stage F-statistics, we think this is unlikely to have had a major effect on our results. In addition, there is a partial overlap in studies that contributed to both GWAS (i.e. some cohort studies have contributed both to GWAS of exposure and also of outcomes). Of the 38 studies included in the CARDIoGRAMplusC4D, 24 appear in GIANT (about 30% of participant overlap) [47]. In the case of weak instruments, the sample overlap between the exposure- and outcome-consortia could bias two-sample MR estimates towards the confounded association between the exposure and the outcome [16, 47]. Nevertheless, as we used genetic instruments strongly associated with our exposure, as suggested by large F-statistics, it is unlikely that our results were biased by weak instruments [47].

In conclusion, our results support a causal effect of higher BMI on CHD risk that is, at least partially, mediated through the effect of BMI on triacylglycerols, HbA_1c_ and type 2 diabetes. These findings support the need for interventional studies examining whether lowering triacylglycerols or providing glucose-lowering therapy for people who are overweight or obese is effective at reducing their increased risk (in comparison with people of healthy weight) of CHD.

## Electronic supplementary material

ESM (PDF 1204 kb)

## Abbreviations

CARDIoGRAM: Coronary ARtery DIsease Genome wide Replication And Meta-analysis
DIAGRAM: DIAbetes Genetics Replication And Meta-analysis
GIANT: Genetic Investigation of ANthropometric Traits
GLGC: Global lipids genetics consortium
GWAS: Genome-wide associations studies
IVW: Inverse variance weighted
MAGIC: Meta-analyses of glucose and insulin-related traits consortium
MR: Mendelian randomisation
