# Supplementary material for: The role of glycaemic and lipid risk factors in mediating the effect of BMI on coronary heart disease: a two-step, two-sample Mendelian randomisation study
Source: Diabetologia. 2017 Sep 9;60(11):2210–20. doi: 10.1007/s00125-017-4396-y (PMC6342872; doi:10.1007/s00125-017-4396-y)
Supplement: Supplementary file 1 — (PDF 1204 kb) [file 125_2017_4396_MOESM1_ESM.pdf]

Electronic supplementary material (ESM)

ESM methods

(1) Details of each of the three two-sample MR methods that were used

In method 1, which we considered to be the main analysis (with other methods providing important sensitivity analyses), the SNP-specific Wald ratio estimates (e.g. the ratio of log odds of CHD per effect allele to change in BMI per effect allele) were combined using the inverse-variance weighted (IVW) approach. This method may be biased if any of the instrumental variables are invalid (i.e. if they influence the outcome by other pathways that are independent from the exposure of interest, known as horizontal pleiotropy).[1] Thus, we also used weighted median (method 2) and MR-Egger (method 3) [2] to combine the SNP specific estimates. These methods seek to obtain an MR estimate that is robust to horizontal pleiotropy. The different assumptions of each method are described as below.

Inverse Variance Weighted (IVW) Method

The IVW method combines the SNP-specific Wald estimates (ratio of SNP on outcome to SNP on exposure) using the following formulas:

$$\hat{\beta}_{IVW} = \frac{\sum_{K=1}^{K} E_k D_k \sigma_{Dk}^{-2}}{\sum_{K=1}^{K} E_k^2 \sigma_{Dk}^{-2}} \text{ (Equation 1)}$$

$$SE_{\hat{\beta}_{IVW}} = \sqrt{\frac{1}{\sum_{k=1}^{k} E_K^2 \sigma_{Dk}^{-2}}} \text{ (Equation 2)}$$

Where E_K is the mean change in exposure level per additional effect allele of SNP k and D_k is the mean change in disease outcomes (e.g. log odds of CHD or levels of other CVD risk factors) per additional effect allele of SNP k with standard error σ_{Dk}. The results using IVW were also used to estimate the intermediating effect of cardiovascular disease risk factors on the causal association between BMI and CHD.

This method provides a consistent estimate of the causal effect if all genetic variants (SNPs) used as instrumental variables satisfy the instrumental variable (IV) assumptions, specifically, 1) the genetic variants are predictive of the exposure, 2) the genetic variants are independent of any confounding factors of the exposure-outcome association, and 3) the genetic variants are independent of the outcome given the exposure and confounding factors (exclusion restriction criteria). It does not test for or take account of horizontal pleiotropy, which if present would result in violation of the exclusion restriction criteria assumption and could importantly bias the estimate of causal effect.

The IVW estimate is a statistically efficient method, but can be biased even if just one genetic variant is invalid (i.e. if just one variant has horizontal pleiotropic effects). For this reason, we used weighted median method in addition to the IVW to account for the possibility of the existence of invalid genetic IVs.

Weighted median estimator

The weighted median estimator is a modification of the simple median approach and in comparison to that approach takes account of the variance of the individual genetic instruments. Specifically, the weighted median estimator is the median of a distribution having estimate β_j as its $P_j = 100(S_j - W_j/2)^{th}$ percentile, where P is the percentile for the jth ordered ratio estimate, w_j is the weight given to the jth ordered ratio estimate, proportional to the inverse of the IV variance, and S_j is the sum of weights up to and including the weight of

the jth ordered ratio estimates, calculated using the following equation.

$\text{Sj} = \sum_{K=1}^{j} W_k$ (Equation 3)

Weights are standardized, so that the sum of the weights Sj is one. As with the simple median, this method assumes that no more than 50% of the genetic IVs are invalid. Additionally it assumes that no single IV contributes more than 50% of the weight. It is more statistically efficient than the simple median method.

MR-Egger regression

The MR-Egger method was developed by Bowden et al. to specifically test for horizontal pleiotropy and correct for this in MR analyses.[1] MR Egger uses a weighted linear regression of the gene–outcome coefficients θ_j on the gene–exposure coefficients δ_j: $\theta_j = \beta_{0E} + \beta_E * \delta_j$, in which all the δ_j associations are orientated to be positive, and the weights in the regression are the inverse-variances of the gene–outcome associations ($\sigma_{Y_j}^{-2}$). If the intercept in the regression model in MR-Egger were truly zero (or were constrained to be zero), the MR-Egger slope estimate β_E is the same as the β from IVW. If the intercept (β_{0E}) is zero it suggests that there is no violation of the exclusion restriction criteria (there is no horizontal pleiotropy); it provides an estimate of the average pleiotropic effect across all of the genetic variants, because it reflects the effect of the joint instruments on outcome (e.g., CHD) when there is zero effect of the genetic variants on the risk factor (e.g. LDL-C). An intercept term that differs from zero suggests horizontal pleiotropy and that the IVW estimate may be biased.

Furthermore, the MR-Egger method provides consistent estimates (from the MR-Egger slope) for the true casual effect even if all genetic variants are invalid due to horizontal pleiotropy (i.e., it represents the causal MR effect having controlled for any violation of the exclusion restriction criteria). However, it requires an additional assumption known as the InSIDE (instrument strength independent of direct effect), which requires that genetic effects on the outcome are direct and not via confounder(s). If the pleiotropic effects of genetic variants are all via a single confounder, they will be correlated with instrument strength, and thus the InSIDE assumption will be violated.

(2) Calculation of the proportion of mediation effect

The proportion of the effect that is mediated by any of the potential mediators was estimated using the following equation [3]:

$$E\ (\%) = \frac{\sum_{K=1}^{K} \beta 1 * \beta 2_k}{\sum_{K=1}^{K} \beta 3 + \beta 1 * \beta 2_k}$$

Where the regression coefficients β1 are the MR effects of BMI on mediator (e.g. triglycerides), β2 the MR effect of mediator k with CHD adjusted for genetically determined BMI, and β3 the MR effect of BMI on CHD adjusted for genetically determined potential mediator. All regression coefficients were derived from MR instrumental analysis using IVW, assuming no correlation between the mediators.

REFERENCES

[1] Bowden J, Davey Smith G, Haycock PC, Burgess S (2016) Consistent Estimation in Mendelian Randomization with Some Invalid Instruments Using a Weighted Median Estimator. Genet Epidemiol 40: 304-314

[2] Bowden J, Davey Smith G, Burgess S (2015) Mendelian randomization with invalid instruments: effect estimation and bias detection through Egger regression. Int J Epidemiol 44: 512-525

[3] Varbo A, Benn M, Smith GD, Timpson NJ, Tybjaerg-Hansen A, Nordestgaard BG (2015) Remnant cholesterol, low-density lipoprotein cholesterol, and blood pressure as mediators from obesity to ischemic heart disease. Circ Res 116: 665-673

[4] Hagg S, Fall T, Ploner A, Magi R, Fischer K, Draisma HH, Kals M, de Vries PS, Dehghan A, Willems SM, Sarin AP, Kristiansson K, Nuotio ML, Havulinna AS, de Bruijn RF, Ikram MA, Kuningas M, Stricker BH, Franco OH, Benyamin B, Gieger C, Hall AS, Huikari V, Jula A, Jarvelin MR, Kaakinen M, Kaprio J, Kobl M, Mangino M, Nelson CP, Palotie A, Samani NJ, Spector TD, Strachan DP, Tobin MD, Whitfield JB, Uitterlinden AG, Salomaa V, Syvanen AC, Kuulasmaa K, Magnusson PK, Esko T, Hofman A, de Geus EJ, Lind L, Giedraitis V, Perola M, Evans A, Ferrieres J, Virtamo J, Kee F, Tregouet DA, Arveiler D, Amouyel P, Gianfagna F, Brambilla P, Ripatti S, van Duijn CM, Metspalu A, Prokopenko I, McCarthy MI, Pedersen NL, Ingelsson E, European Network for G, Genomic Epidemiology C (2015) Adiposity as a cause of cardiovascular disease: a Mendelian randomization study. Int J Epidemiol 44: 578-586

[5] Holmes MV, Lange LA, Palmer T, Lanktree MB, North KE, Almoguera B, Buxbaum S, Chandrupatla HR, Elbers CC, Guo Y, Hoogeveen RC, Li J, Li YR, Swerdlow DI, Cushman M,

Price TS, Curtis SP, Fornage M, Hakonarson H, Patel SR, Redline S, Siscovick DS, Tsai MY, Wilson JG, van der Schouw YT, FitzGerald GA, Hingorani AD, Casas JP, de Bakker PI, Rich SS, Schadt EE, Asselbergs FW, Reiner AP, Keating BJ (2014) Causal effects of body mass index on cardiometabolic traits and events: a Mendelian randomization analysis. Am J Hum Genet 94: 198-208

[6] Nordestgaard BG, Palmer TM, Benn M, Zacho J, Tybjærg-Hansen A, Davey Smith G, Timpson NJ (2012) The Effect of Elevated Body Mass Index on Ischemic Heart Disease Risk: Causal Estimates from a Mendelian Randomisation Approach. PLoS Med 9: e1001212

ESM Table 1: Characteristics of the single nucleotide polymorphisms (SNP) used as instrumental variables for body mass index (kg/m2)[b].

SNP	Gene	Effect allele	Other allele	EAF	Effect[a]	Standard error	P-value
rs1558902	*FTO*	A	T	0.415	0.082	0.003	7.51E-153
rs6567160	*MC4R*	C	T	0.236	0.056	0.004	3.93E-53
rs13021737	*TMEM18*	G	A	0.828	0.06	0.004	1.11E-50
rs10938397	*GNPDA2 GABRG1*	G	A	0.434	0.04	0.003	3.21E-38
rs543874	*SEC16B*	G	A	0.193	0.048	0.004	2.62E-35
rs2207139	*TFAP2B*	G	A	0.177	0.045	0.004	4.13E-29
rs11030104	*BDAF*	A	G	0.792	0.041	0.004	5.56E-28
rs3101336	N*EGR1*	C	T	0.613	0.033	0.003	2.66E-26
rs7138803	*BCDIN3D FAIM2*(D)	A	G	0.384	0.032	0.003	8.15E-24
rs10182181	*ADCY3 POMC*(B,G); *NCOA1 SH2B1*(B,M,Q); *APOBR*(M,Q);	G	A	0.462	0.031	0.003	8.78E-24
rs3888190	*ATXN2L SBK1*(Q,D); *SULT1A2* ; *TUFM*	A	C	0.403	0.031	0.003	3.14E-23
rs1516725	*E7V5*	C	T	0.872	0.045	0.005	1.89E-22
rs12446632	*GPRC5BIQCK*	G	A	0.865	0.04	0.005	1.48E-18
rs2287019	*QPCTL* ; *GIPR*	C	T	0.804	0.036	0.004	4.59E-18
rs16951275	*M4P2K5 LBXCOR1*(M)	T	C	0.784	0.031	0.004	1.91E-17
rs3817334	*MTCH2C1QTNF4*(Q,I); *SPI1* ; *CELF1*(D)	T	C	0.407	0.026	0.003	5.15E-17
rs2112347	*POC5*; *HMGCR COL4A3BP*	T	G	0.629	0.026	0.003	6.19E-17
rs12566985	*FPGT-TNNI3K*	G	A	0.446	0.024	0.003	3.28E-15
rs3810291	*ZC3H4*	A	G	0.666	0.028	0.004	4.81E-15
rs7141420	*NRXN3*	T	C	0.527	0.024	0.003	1.23E-14
rs13078960	*CADM2*	G	T	0.196	0.03	0.004	1.74E-14
rs10968576	*LINGO2*	G	A	0.32	0.025	0.003	6.61E-14
rs17024393	*GNAT2* ; *AMPD2*	C	T	0.04	0.066	0.009	7.03E-14
rs12429545	*OLFM4*	A	G	0.133	0.033	0.005	1.09E-12
rs13107325	*SLC39A8*	T	C	0.072	0.048	0.007	1.83E-12
rs11165643	*PTBP2*	T	C	0.583	0.022	0.003	2.07E-12
rs17405819	*HNF4G*	T	C	0.7	0.022	0.003	2.07E-11
rs1016287	*LINC01122*	T	C	0.287	0.023	0.003	2.25E-11
rs4256980	*TRIM66 TUB*	G	C	0.646	0.021	0.003	2.90E-11
rs12401738	*FUBP1* ; *USP33*	A	G	0.352	0.021	0.003	1.15E-10
rs205262	*C6orf106* ; *SNRPC*	G	A	0.273	0.022	0.004	1.75E-10
rs12016871	*MTIF3* ; *GTF3A*	T	C	0.203	0.03	0.005	2.29E-10
rs12940622	*RPTOR*	G	A	0.575	0.018	0.003	2.49E-09
rs11847697	*PRKD1*	T	C	0.042	0.049	0.008	3.99E-09
rs2075650	*TOMM40 APOE APOC1*	A	G	0.848	0.026	0.005	1.25E-08
rs2121279	*LRP1B*	T	C	0.152	0.025	0.004	2.31E-08
rs29941	*KCTD15*	G	A	0.669	0.018	0.003	2.41E-08

rs1808579	*NPC1*; *C18orf8*(N,Q)	C	T	0.534	0.017	0.003	4.17E-08
rs657452	*AGBL4*	A	G	0.394	0.023	0.003	5.48E-13
rs12286929	*CADM1*	G	A	0.523	0.022	0.003	1.31E-12
rs7903146	*TCF7L2*	C	T	0.713	0.023	0.003	1.11E-11
rs10132280	*STXBP6*	C	A	0.682	0.023	0.003	1.14E-11
rs17094222	*HIF1AN*	C	T	0.211	0.025	0.004	5.94E-11
rs7599312	*ERBB4*	G	A	0.724	0.022	0.003	1.17E-10
rs2365389	*FHIT*	C	T	0.582	0.020	0.003	1.63E-10
rs2820292	*NAV1*	C	A	0.555	0.020	0.003	1.83E-10
rs12885454	*PRKD1*	C	A	0.642	0.021	0.003	1.94E-10
rs16851483	*RASA2*	T	G	0.066	0.048	0.008	3.55E-10
rs1167827	*HIP1* ; *PMS2L3* *PMS2P5* ;		A	0.553	0.020	0.003	6.33E-10
	WBSCR16	G					
rs758747	*NLRC3*	T	C	0.265	0.023	0.004	7.47E-10
rs1928295	*TLR4*	T	C	0.548	0.019	0.003	7.91E-10
rs9925964	*KAT8* ;*ZNF646* *VKORC1* ;		G	0.620	0.019	0.003	8.11E-10
	ZNF668 ; *STX1B* *FBXL19*(D)	A					
rs11126666	*KCNK3*	A	G	0.283	0.021	0.003	1.33E-09
rs2650492	*SBK1* ; *APOBR*	A	G	0.303	0.021	0.004	1.92E-09
rs6804842	*RARB*	G	A	0.575	0.019	0.003	2.48E-09
rs4740619	*C9orf93*	T	C	0.542	0.018	0.003	4.56E-09
rs13191362	*PARK2*	A	G	0.879	0.028	0.005	7.34E-09
rs3736485	*SCG3*; *DMXL2*(M,N)	A	G	0.454	0.018	0.003	7.41E-09
rs17001654	*NUP54*; *SCARB2*(Q,N)	G	C	0.153	0.031	0.005	7.76E-09
rs11191560	*NT5C2* ; *CYP17A1* *SFXN2*	C	T	0.089	0.031	0.005	8.45E-09
rs1528435	*UBE2E3*	T	C	0.631	0.018	0.003	1.20E-08
rs1000940	*RABEP1*	G	A	0.320	0.019	0.003	1.28E-08
rs2033529	*TDRG1* ; *LRFN2*	G	A	0.293	0.019	0.003	1.39E-08
rs11583200	*ELAVL4*	C	T	0.396	0.018	0.003	1.48E-08
rs9400239	*FOXO3* *HSS00296402*	C	T	0.688	0.019	0.003	1.61E-08
rs10733682	*LMX1B*	A	G	0.478	0.017	0.003	1.83E-08
rs11688816	*EHBP1*	G	A	0.525	0.017	0.003	1.89E-08
rs11057405	*CLIP1*	G	A	0.901	0.031	0.006	2.02E-08
rs11727676	*HHIP*	T	C	0.910	0.036	0.006	2.55E-08
rs3849570	*GBE1*	A	C	0.359	0.019	0.003	2.60E-08
rs6477694	*EPB41L4B* ; *C9orf4*(D)	C	T	0.365	0.017	0.003	2.67E-08
rs7899106	*GRID1*	G	A	0.052	0.040	0.007	2.96E-08
rs2176598	*HSD17B12*	T	C	0.251	0.020	0.004	2.97E-08
rs2245368	*PMS2L11*	C	T	0.180	0.032	0.006	3.19E-08
rs17724992	*GDF15* *PGPEP1*(Q,N)	A	G	0.746	0.019	0.004	3.42E-08
rs7243357	*GRP*	T	G	0.812	0.022	0.004	3.86E-08
rs2033732	*RALYL*	C	T	0.747	0.019	0.004	4.89E-08
The following 20 SNPs identified from the secondary analysis were used in the sensitivity analysis							
rs9641123	*CALCR* ; *hsa-miR*-653	C	G	0.430	0.029	0.005	2.08E-10

rs7164727	*LOC100287559* , *BBS4*(B,M,Q)	T	C	0.671	0.019	0.003	3.92E-09
rs492400	*PLCD4* *CYP27A1* USP37 ; *TTLL4*(M,Q); *STK36*(B,M); *ZNF142*(M); *RQCD1*	C	T	0.424	0.024	0.004	6.78E-09
rs2080454	*CBLN1*	C	A	0.413	0.017	0.003	8.60E-09
rs7239883	*LOC284260* ; *RIT2*(B,D)	G	A	0.391	0.023	0.004	1.51E-08
rs2836754	*ETS2*	C	T	0.599	0.017	0.003	1.61E-08
rs9914578	*SMG6* ; *N29617*	G	C	0.229	0.020	0.004	2.07E-08
rs977747	*TAL1*	T	G	0.403	0.017	0.003	2.18E-08
rs9374842	*LOC285762* ;	T	C	0.744	0.023	0.004	2.67E-08
rs4787491	*MAPK3* *KCTD13* *INO80E* ; *TAOK2* *YPEL3* *DOC2A* *FAM57B*(D)	G	A	0.510	0.022	0.004	2.70E-08
rs1441264	*MIR548A2*	A	G	0.613	0.017	0.003	2.96E-08
rs17203016	*CREB1* ; *KLF7*	G	A	0.195	0.021	0.004	3.41E-08
rs16907751	*ZBTB10*	C	T	0.913	0.047	0.009	3.89E-08
rs13201877	*IFNGR1* ; *OLIG3*	G	A	0.140	0.024	0.004	4.29E-08
rs9540493	*MIR548X2* ; *PCDH9*(D)	A	G	0.452	0.021	0.004	4.97E-08
rs1460676	*FIGN*	C	T	0.179	0.021	0.004	4.98E-08
rs6465468	*ASB4*	T	G	0.306	0.025	0.005	4.98E-08
rs6091540	*ZFP64*	C	T	0.721	0.030	0.004	2.15E-11
rs7715256	*GALNT10*	G	T	0.422	0.017	0.003	8.85E-09
rs2176040	*LOC646736* ; *IRS1*(B,Q)	A	G	0.365	0.024	0.004	9.99E-09

EAF: effect allele frequency

[a]: Increase in body mass index (kg/m^2) per effect allele

[b]: The variants together explain 2.7% of the variation in BMI (R^2 =0.027).

Source of data: Locke AE, Kahali B, Berndt SI, et al. Genetic studies of body mass index yield new insights for obesity biology. Nature. 2015;518(7538):197-206.

ESM Table 2: Characteristics of the single nucleotide polymorphisms (SNP) used as instrumental variables for triglycerides[b] (SD, 1 SD= 1.024mmol/l).

SNP	Gene	Effect allele	Other allele	EAF	Effect[a]	P-value
ANGPTL3	rs2131925	0.34	G	T	-0.066	3.00E-74
GCKR	rs1260326	0.39	T	C	0.115	2.00E-239
MSL2L1	rs645040	0.23	G	T	-0.029	2.00E-12
KLHL8	rs442177	0.42	G	T	-0.031	1.00E-18
MAP3K1	rs9686661	0.20	T	C	0.038	3.00E-16
TYW1B	rs13238203	0.04	T	C	-0.059	3.00E-06
MLXIPL	rs17145738	0.13	T	C	-0.115	9.00E-99
PINX1	rs11776767	0.37	C	G	0.022	3.00E-11
NAT2	rs1495741	0.26	G	A	0.040	3.00E-12
LPL	rs12678919	0.13	G	A	-0.170	2.00E-199
TRIB1	rs2954029	0.47	T	A	-0.076	1.00E-107
JMJD1C	rs10761731	0.44	T	A	-0.031	8.00E-12
CYP26A1	rs2068888	0.45	A	G	-0.024	2.00E-11
FADS1-2-3	rs174546	0.36	T	C	0.045	7.00E-38
APOA1	rs964184	0.84	C	G	-0.234	7.00E-224
LRP1	rs11613352	0.26	T	C	-0.028	9.00E-14
CAPN3	rs2412710	0.04	A	G	0.099	2.00E-11
FRMD5	rs2929282	0.07	T	A	0.072	2.00E-09
CTF1	rs11649653	0.40	G	C	-0.027	2.00E-07
PLA2G6	rs5756931	0.40	C	T	-0.020	3.00E-08
LRPAP1	rs6831256	0.42	G	A	0.026	2.00E-12
VEGFA	rs998584	0.49	A	C	0.029	3.00E-15
MET	rs38855	0.47	G	A	-0.019	2.00E-08
AKR1C4	rs1832007	0.18	G	A	-0.033	2.00E-12
PDXDC1	rs3198697	0.43	T	C	-0.020	2.00E-08
MPP3	rs8077889	0.22	C	A	0.025	1.00E-08
INSR	rs7248104	0.42	A	G	-0.022	5.00E-10
PEPD	rs731839	0.35	G	A	0.022	3.00E-09

EAF: effect allele frequency

[a]: Increase in triglycerides (SD, 1 SD= 1.024 mmol/l) per effect allele

[b]: The variants together explain 2.1% of the variation in triglycerides levels (R^2 =0.021).

Source of data: Global Lipids Genetics C, Willer CJ, Schmidt EM, et al. Discovery and refinement of loci associated with lipid levels. Nat Genet. 2013;45(11):1274-1283.

ESM Table 3: Characteristics of the single nucleotide polymorphisms (SNP) used as instrumental variables for high-density lipoprotein (HDL) cholesterol[b] (SD, 1 SD= 0.4 mmol/l).

SNP	Gene	Effect allele	Other allele	EAF	Effect[a]	P-value
PABPC4	rs4660293	0.24	G	A	-0.035	3.00E-18
ZNF648	rs1689800	0.35	G	A	-0.034	5.00E-20
GALNT2	rs4846914	0.41	G	A	-0.048	4.00E-41
COBLL1	rs12328675	0.13	C	T	0.045	2.00E-15
IRS1	rs2972146	0.37	G	T	0.032	2.00E-17
SLC39A8	rs13107325	0.08	T	C	-0.071	1.00E-15
ARL15	rs6450176	0.26	A	G	-0.025	7.00E-10
CITED2	rs605066	0.42	C	T	-0.028	3.00E-08
KLF14	rs4731702	0.49	T	C	0.029	5.00E-17
PPP1R3B	rs9987289	0.10	A	G	-0.082	2.00E-41
TRPS1	rs2293889	0.41	T	G	-0.031	4.00E-17
TTC39B	rs581080	0.21	G	C	-0.042	1.00E-19
ABCA1	rs1883025	0.25	T	C	-0.07	2.00E-65
AMPD3	rs2923084	0.18	G	A	-0.026	5.00E-08
LRP4	rs3136441	0.18	C	T	0.054	7.00E-29
PDE3A	rs7134375	0.43	A	C	0.021	1.00E-08
MVK	rs7134594	0.48	C	T	-0.035	2.00E-13
SBNO1	rs4759375	0.08	T	C	0.056	3.00E-08
ZNF664	rs4765127	0.35	T	G	0.032	8.00E-10
SCARB1	rs838880	0.34	C	T	0.048	6.00E-32
LIPC	rs1532085	0.40	A	G	0.107	1.00E-188
LACTB	rs2652834	0.21	A	G	-0.028	4.00E-11
CETP	rs3764261	0.32	A	C	0.241	1E-769
LCAT	rs16942887	0.14	A	G	0.083	8.00E-54
CMIP	rs2925979	0.31	T	C	-0.035	1.00E-19
STARD3	rs11869286	0.35	G	C	-0.032	3.00E-17
ABCA8	rs4148008	0.33	G	C	-0.028	1.00E-12
PGS1	rs4129767	0.48	G	A	-0.024	2.00E-11
LIPG	rs7241918	0.19	G	T	-0.09	1.00E-44

MC4R	rs12967135	0.25	A	G	-0.026	4.00E-08
ANGPTL4	rs7255436	0.47	C	A	-0.032	2.00E-08
ANGPTL8	rs737337	0.11	C	T	-0.056	5.00E-17
LILRA3	rs386000	0.26	C	G	0.048	3.00E-23
HNF4A	rs1800961	0.05	T	C	-0.127	2.00E-34
PLTP	rs6065906	0.19	C	T	-0.059	5.00E-40
UBE2L3	rs181362	0.23	T	C	-0.038	4.00E-18
PIGV-NR0B2	rs12748152	0.09	T	C	-0.051	1.00E-15
HDGF-PMVK	rs12145743	0.34	G	T	0.020	2.00E-08
ANGPTL1	rs4650994	0.49	G	A	0.021	7.00E-09
CPS1	rs1047891	0.33	A	C	-0.027	9.00E-10
ATG7	rs2606736	0.39	C	T	0.025	5.00E-08
SETD2	rs2290547	0.2	A	G	-0.030	4.00E-09
RBM5	rs2013208	0.5	T	C	0.025	9.00E-12
STAB1	rs13326165	0.21	A	G	0.029	9.00E-11
GSK3B	rs6805251	0.39	T	C	0.020	1.00E-08
C4orf52	rs10019888	0.18	G	A	-0.027	5.00E-08
FAM13A	rs3822072	0.46	A	G	-0.025	4.00E-12
ADH5	rs2602836	0.44	A	G	0.019	5.00E-08
RSPO3	rs1936800	0.49	C	T	0.020	3.00E-10
DAGLB	rs702485	0.45	G	A	0.024	6.00E-12
SNX13	rs4142995	0.38	T	G	-0.026	9.00E-12
IKZF1	rs4917014	0.32	G	T	0.022	1.00E-08
TMEM176A	rs17173637	0.12	C	T	-0.036	2.00E-08
MARCH8-ALOX5	rs970548	0.26	C	A	0.026	2.00E-10
OR4C46	rs11246602	0.15	C	T	0.034	2.00E-10
KAT5	rs12801636	0.23	A	G	0.024	3.00E-08
MOGAT2-DGAT2	rs499974	0.19	A	C	-0.026	1.00E-08
ZBTB42-AKT1	rs4983559	0.4	G	A	0.020	1.00E-08
FTO	rs1121980	0.43	A	G	-0.020	7.00E-09
HAS1	rs17695224	0.26	A	G	-0.029	2.00E-13

EAF: effect allele frequency;

[a]: Increase in HDL-cholesterol (SD, 1 SD= 0.4 mmol/l) per effect allele

[b]: The variants together explain 1.6% of the variation in HDL-cholesterol levels (R^2 =0.016).

Source of data: Global Lipids Genetics C, Willer CJ, Schmidt EM, et al. Discovery and refinement of loci associated with lipid levels. Nat Genet. 2013;45(11):1274-1283.

ESM Table 4: Characteristics of the single nucleotide polymorphisms (SNP) used as instrumental variables for low-density lipoprotein (LDL) cholesterol[b] (SD, 1 SD= 1.0 mmol/l).

SNP	Gene	Effect allele	Other allele	EAF	Effect[a]	P-value
rs2479409	*PCSK9*	G	A	0.32	0.064	3.00E-50
rs629301	*SORT1*	G	T	0.24	-0.167	5.00E-241
rs1367117	*APOB*	A	G	0.32	0.119	1.00E-182
rs4299376	*ABCG5/8*	G	T	0.31	0.081	4.00E-72
rs3757354	*MYLIP*	T	C	0.24	-0.038	2.00E-17
rs1800562	*HFE*	A	G	0.07	-0.062	8.00E-14
rs1564348	*LPA*	C	T	0.18	0.048	3.00E-21
rs11136341	*PLEC1*	G	A	0.40	0.045	7.00E-12
rs9411489	*ABO*	T	C	0.21	0.077	2.00E-41
rs11220462	*ST3GAL4*	A	G	0.14	0.059	7.00E-21
rs8017377	*NYNRIN*	A	G	0.46	0.030	3.00E-15
rs7206971	*OSBPL7*	A	G	0.49	0.029	3.00E-07
rs6511720	*LDLR*	T	G	0.12	-0.221	4.00E-262
rs4420638	*APOE*	G	A	0.19	0.225	2.00E-178
rs6029526	*TOP1*	A	T	0.47	0.044	5.00E-18
rs267733	*ANXA9-CERS2*	G	A	0.16	-0.033	5.00E-09
rs2710642	*EHBP1*	G	A	0.35	-0.024	6.00E-09
rs10490626	*INSIG2*	A	G	0.08	-0.051	2.00E-12
rs2030746	*LOC84931*	T	C	0.4	0.021	9.00E-09
rs1250229	*FN1*	T	C	0.27	-0.024	3.00E-08
rs7640978	*CMTM6*	T	C	0.09	-0.039	1.00E-08
rs17404153	*ACAD11*	T	G	0.14	-0.034	2.00E-09
rs4530754	*CSNK1G3*	G	A	0.46	-0.028	4.00E-12
rs4722551	*MIR148A*	C	T	0.2	0.039	4.00E-14
rs10102164	*SOX17*	A	G	0.21	0.032	4.00E-11
rs4942486	*BRCA2*	T	C	0.48	0.024	2.00E-11
rs1801689	*APOH-PRXCA*	C	A	0.04	0.103	1.00E-11
rs364585	*SPTLC3*	A	G	0.38	-0.025	4.00E-10
rs2328223	*SNX5*	C	A	0.21	0.030	6.00E-09

rs5763662	*MTMR3*	T	C	0.04	0.077	1.00E-08

EAF: effect allele frequency;

[a]: Increase in LDL-cholesterol (SD, 1 SD= 1.0 mmol/l) per effect allele

[b] The variants together explain 2.4% of the variation in LDL cholesterol levels (R^2 =0.024).

Source of data: Global Lipids Genetics C, Willer CJ, Schmidt EM, et al. Discovery and refinement of loci associated with lipid levels. Nat Genet. 2013;45(11):1274-1283.

ESM Table 5: Characteristics of the single nucleotide polymorphisms (SNP) used as instrumental variables for fasting glucose[b] (mmol/l).

SNP	Gene	Effect allele	Other allele	EAF	Effect[a]	SE	P-value
rs10747083	*P2RX2*	A	G	0.25	0.013	0.0023	7.57E-09
rs10811661	*CDKN2B*	T	C	0.199	0.024	0.0028	5.65E-18
rs10814916	*GLIS3*	C	A	0.434	0.016	0.0022	2.26E-13
rs10830963	*MTNR1B*	C	G	0.3	-0.078	0.0025	1.07E-215
rs10885122	*ADRA2A*	G	T	0.1	0.027	0.0033	6.32E-17
rs11039182	*MADD*	T	C	0.308	0.023	0.0024	4.82E-22
rs11195502	*ADRA2A*	C	T	0.075	0.032	0.0037	1.97E-18
rs11558471	*GCK*	A	G	0.252	0.029	0.0023	7.80E-37
rs11603334	*ARAP1*	G	A	0.129	0.019	0.0028	1.12E-11
rs11605924	*CRY2*	A	C	0.458	0.02	0.0023	3.93E-19
rs11607883	*CRY2*	G	A	0.469	0.021	0.0021	6.32E-24
rs11619319	*PDX1*	A	G	0.212	-0.02	0.0024	1.33E-15
rs11708067	*ADCY5*	A	G	0.226	0.023	0.0026	1.30E-18
rs11715915	*AMT*	C	T	0.274	0.012	0.0022	4.90E-08
rs12440695	*VPS13C/C2CD4A/B*	C	T	0.363	-0.01	0.0022	3.89E-06
rs1280	*SLC2A2*	T	C	0.137	0.026	0.0031	8.56E-18
rs16913693	*IKBKAP*	T	G	0.017	0.043	0.0066	3.51E-11
rs174550	*FADS1*	T	C	0.367	0.019	0.0022	1.34E-17
rs174576	*FADS1*	C	A	0.345	0.02	0.0022	1.18E-18
rs17762454	*RREB1*	C	T	0.217	-0.012	0.0023	1.88E-07
rs2191349	*DGKB/TMEM195*	G	T	0.467	-0.029	0.0021	1.28E-42
rs2302593	*GIPR*	C	G	0.475	0.014	0.0023	9.26E-10
rs2657879	*GLS2*	A	G	0.186	-0.012	0.0027	5.69E-06
rs2908289	*GCK*	G	A	0.2	-0.057	0.0029	3.32E-88
rs340874	*PROX1*	C	T	0.438	0.013	0.0022	4.08E-10
rs3783347	*WARS*	G	T	0.219	0.017	0.0026	1.32E-10
rs3829109	*DNLZ*	G	A	0.345	0.017	0.0027	1.13E-10
rs4502156	*VPS13C/C2CD4A/B*	T	C	0.42	0.022	0.0021	1.38E-25
rs4506565	*TCF7L2*	A	T	0.296	-0.021	0.0023	3.95E-19

rs4869272	*PCSK1*	C	T	0.323	-0.018	0.0022	1.02E-15
rs560887	*G6PC2*	C	T	0.326	0.071	0.0025	1.40E-178
rs576674	*KL*	G	A	0.129	0.017	0.003	2.26E-08
rs6072275	*TOP1*	G	A	0.142	-0.016	0.0028	1.66E-08
rs6113722	*FOXA2*	G	A	0.04	0.035	0.0053	2.49E-11
rs6943153	*GRB10*	C	T	0.279	-0.015	0.0022	1.63E-12
rs730497	*GCK*	G	A	0.195	-0.057	0.0029	3.70E-87
rs7651090	*IGF2BP2*	A	G	0.296	-0.013	0.0023	1.75E-08
rs7708285	*ZBED3*	G	A	0.261	0.011	0.0025	4.89E-06
rs780094	*GCKR*	C	T	0.394	0.027	0.0021	2.58E-37
rs7867224	*GLIS3*	A	G	0.466	0.013	0.0023	3.90E-09
rs7903146	*TCF7L2*	C	T	0.279	-0.022	0.0024	2.71E-20
rs9368222	*CDKAL1*	C	A	0.281	-0.014	0.0023	1.00E-09
rs983309	*PPP1R3B*	G	T	0.097	-0.026	0.0033	6.29E-15

EAF: effect allele frequency;

[a:] Increase in fasting glucose (mmol/l) per effect allele

[b]: The variants together explain 4.8% of the variation in fasting glucose levels (R^2 =0.048).

Source of data: Scott RA, Lagou V, Welch RP, et al. Large-scale association analyses identify new loci influencing glycemic traits and provide insight into the underlying biological pathways. Nat Genet. 2012;44(9):991-1005.

ESM Table 6: Characteristics of the single nucleotide polymorphisms (SNP) used as instrumental variables for fasting insulin[b] (log-pmol/l).

SNP	Gene	Effect allele	Other allele	EAF	Effect[a]	SE	P-value
rs10195252	*GRB14*	C	T	0.442	-0.016	0.0026	4.87E-10
rs1167800	*HIP1*	A	G	0.451	0.016	0.0026	2.61E-09
rs1421085	*FTO*	T	C	0.46	-0.02	0.0025	1.87E-15
rs1530559	*YSK4*	A	G	0.403	0.014	0.0026	3.37E-08
rs17036328	*PPARG*	T	C	0.097	0.015	0.0036	1.92E-05
rs2126259	*PPP1R3B*	C	T	0.088	-0.025	0.004	1.47E-10
rs2745353	*RSPO3*	T	C	0.45	0.014	0.0025	5.48E-09
rs2820436	*LYPLAL1*	C	A	0.322	0.015	0.0026	4.36E-09
rs2943645	*IRS1*	C	T	0.372	-0.013	0.0025	1.37E-07
rs2972143	*IRS1*	G	A	0.371	0.014	0.0026	3.15E-08
rs3822072	*FAM13A1*	G	A	0.455	-0.0092	0.0024	1.19E-04
rs459193	*AP3K1*	G	A	0.217	0.014	0.0027	6.57E-08
rs4846565	*LYPLAL1*	G	A	0.31	0.015	0.0026	2.01E-08
rs4865796	*ARL15*	A	G	0.292	0.015	0.0026	2.09E-08
rs6822892	*PDGFC*	A	G	0.354	0.011	0.0025	3.16E-05
rs6912327	*UHRF1BP1*	C	T	0.243	-0.016	0.0034	2.84E-06
rs731839	*PEPD*	A	G	0.341	-0.014	0.0026	1.72E-08
rs974801	*TET2*	G	A	0.394	0.016	0.0025	2.13E-10
rs9884482	*TET2*	T	C	0.35	-0.016	0.0024	1.40E-11

EAF: effect allele frequency;

[a]: Increase in fasting insulin (log pmol/l) per effect allele

[b]: The variants together explain 1.2% of the variation in fasting insulin levels (R^2 =0.012).

Source of data: Scott RA, Lagou V, Welch RP, et al. Large-scale association analyses identify new loci influencing glycemic traits and provide insight into the underlying biological pathways. Nat Genet. 2012;44(9):991-1005.

ESM Table 7: Characteristics of the single nucleotide polymorphisms (SNP) used as instrumental variables for HbA1c[b] (%).

SNP	Gene	Effect allele	Other allele	EAF	Effect[a]	SE	P-value
rs1046896	*FN3K*	T	C	0.31	0.035	0.003	1.57E-26
rs1387153	*MTNR1B*	T	C	0.28	0.028	0.004	3.96E-11
rs16926246	*HK1*	C	T	0.9	0.089	0.004	3.11E-54
rs1799884	*GCK*	T	C	0.18	0.038	0.004	1.45E-20
rs1800562	*HFE*	G	A	0.94	0.063	0.007	2.59E-20
rs2779116	*SPTA1*	T	C	0.27	0.024	0.004	2.75E-09
rs4737009	*ANK1*	A	G	0.24	0.027	0.004	6.11E-12
rs552976	*G6PC2/ABCB11*	G	A	0.64	0.047	0.003	8.16E-18
rs7998202	*ATP11A/TUBGCP3*	G	A	0.14	0.031	0.005	5.24E-09
rs855791	*TMPRSS6*	A	G	0.42	0.027	0.004	2.74E-14

EAF: effect allele frequency;

[a]: Increase in HbA_{1c} (%) per effect allele

[b]: The variants together explain 2.4% of the variation in HbA_{1c} levels (R^2 =0.024).

Source of data: Soranzo N, Sanna S, Wheeler E, et al. Common variants at 10 genomic loci influence hemoglobin A(1)(C) levels via glycemic and nonglycemic pathways. Diabetes. 2010;59(12):3229-3239.

ESM Table 8: Characteristics of the single nucleotide polymorphisms (SNP) used as instrumental variables for type 2 diabetes[b].

SNP	Gene	Effect allele	Other allele	*p*-value	OR (95% CI) [a]
rs2075423	*PROX1*	G	T	8.1E-09	1.07 (1.05, 1.10)
rs10203174	*THADA*	C	T	9.5E-12	1.14 (1.10, 1.19)
rs243088	*BCL11A*	T	A	1.8E-08	1.07 (1.04, 1.09)
rs13389219	*GRB14*	C	T	1.0E-08	1.07 (1.05, 1.10)
rs2943640	*IRS1*	C	A	2.7E-14	1.10 (1.07, 1.12)
rs1801282	*PPARG*	C	G	1.1E-12	1.13 (1.09, 1.17)
rs1496653	*UBE2E2*	A	G	3.6E-09	1.09 (1.06, 1.12)
rs6795735	*ADAMTS9*	C	T	7.4E-11	1.08 (1.06, 1.11)
rs11717195	*ADCY5*	T	C	6.5E-14	1.11 (1.08, 1.14)
rs4402960	*IGF2BP2*	T	G	2.4E-23	1.13 (1.10, 1.16)
rs4458523	*WFS1*	G	T	2.0E-15	1.10 (1.07, 1.12)
rs459193	*ANKRD55*	G	A	6.0E-09	1.08 (1.05, 1.11)
rs6878122	*ZBED3*	G	A	5.0E-11	1.10 (1.07, 1.13)
rs7756992	*CDKAL1*	G	A	7.0E-35	1.17 (1.14, 1.20)
rs17168486	*DGKB*	T	C	5.9E-11	1.11 (1.07, 1.14)
rs849135	*JAZF1*	G	A	3.1E-17	1.11 (1.08, 1.13)
rs516946	*ANK1*	C	T	2.5E-10	1.09 (1.06, 1.12)
rs3802177	*SLC30A8*	G	A	1.3E-21	1.14 (1.11, 1.17)
rs10811661	*CDKN2A/B*	T	C	3.7E-27	1.18 (1.15, 1.22)
rs2796441	*TLE1*	G	A	5.4E-09	1.07 (1.05, 1.10)
rs12571751	*ZMIZ1*	A	G	1.0E-10	1.08 (1.05, 1.10)
rs1111875	*HHEX/IDE*	C	T	2.0E-19	1.11 (1.09, 1.14)
rs7903146	*TCF7L2*	T	C	1.2E-139	1.39 (1.35, 1.42)
rs163184	*KCNQ1*	G	T	1.2E-11	1.09 (1.06, 1.11)
rs5215	*KCNJ11*	C	T	8.5E-10	1.07 (1.05, 1.10)
rs1552224	*ARAP1 (CENTD2)*	A	C	1.8E-10	1.11 (1.07, 1.14)
rs10830963	*MTNR1B*	G	C	5.3E-13	1.10 (1.07, 1.13)
rs10842994	*KLHDC5*	C	T	6.1E-10	1.10 (1.06, 1.13)
rs2261181	*HMGA2*	T	C	1.2E-09	1.13 (1.08, 1.17)
rs7955901	*TSPAN8/LGR5*	C	T	6.5E-09	1.07 (1.05, 1.10)
rs1359790	*SPRY2*	G	A	1.4E-08	1.08 (1.05, 1.10)
rs7177055	*HMG20A*	A	G	4.6E-09	1.08 (1.05, 1.10)
rs12899811	*PRC1*	G	A	6.3E-09	1.08 (1.05, 1.10)
rs9936385	*FTO*	C	T	2.6E-23	1.13 (1.10, 1.16)
rs7202877	*BCAR1*	T	G	3.5E-08	1.12 (1.07, 1.16)
rs11651052	*HNF1B (TCF2)*	A	G	2.0E-11	1.10 (1.07, 1.14)
rs12970134	*MC4R*	A	G	1.2E-08	1.08 (1.05, 1.11)
rs10401969	*CILP2*	C	T	7.0E-09	1.13 (1.09, 1.18)

OR: odds ratio; CI: confidence interval

[a]: Increase in odds of type 2 diabetes with increase in per effect allele.

[b]: The variants together explain 5.7% of the variation in the risk for type 2 diabetes, as calculated by transforming

dichotomous disease risk onto a continuous liability scale (R^2 =0.057).

Source of data: Morris AP, Voight BF, Teslovich TM, et al. Large-scale association analysis provides insights into the genetic architecture and pathophysiology of type 2 diabetes. Nat Genet. 2012;44(9):981-990.

ESM Table 9: Characteristics of the single nucleotide polymorphisms (SNP) used as instrumental variables for coronary heart disease.

SNP	Gene	Effect allele	Other allele	EAF	*p*-value	OR (95% CI) [a]
rs10139550	HHIPL1	G	C	0.423	1.38E-08	1.06 (1.04, 1.08)
rs10840293	SWAP70	A	G	0.55	1.30E-08	1.06 (1.04, 1.08)
rs11191416	CYP17A1-CNNM2-NT5C2	T	G	0.873	4.65E-09	1.08 (1.05, 1.11)
rs11206510	PCSK9	T	C	0.848	2.34E-08	1.08 (1.05, 1.11)
rs11556924	ZC3HC1	C	T	0.687	5.34E-11	1.08 (1.05, 1.10)
rs11830157	KSR2	G	T	0.36	2.12E-09	1.12 (1.08, 1.16)
rs11838776	COL4A1/A2	A	G	0.263	1.83E-10	1.07 (1.05, 1.09)
rs12202017	TCF21*	A	G	0.7	1.98E-11	1.07 (1.05, 1.09)
rs12976411	ZNF507-LOC400684	T	A	0.09	1.18E-14	0.67 (0.60, 0.74)
rs1412444	LIPA	T	C	0.369	5.15E-12	1.07 (1.05, 1.09)
rs16986953	AK097927	A	G	0.105	1.45E-08	1.09 (1.06, 1.12)
rs17087335	REST-NOA1	T	G	0.21	4.60E-08	1.06 (1.04, 1.09)
rs17678683	ZEB2-ACO74093.1	G	T	0.088	3.00E-09	1.10 (1.07, 1.14)
rs180803	POM121L9P-ADORA2A	G	T	0.97	1.60E-10	1.20 (1.13, 1.27)
rs1870634	CXCL12	G	T	0.637	5.55E-15	1.08 (1.06, 1.10)
rs2107595	HDAC9	A	G	0.2	8.05E-11	1.08 (1.05, 1.10)
rs2128739	PDGFD	A	C	0.324	7.05E-11	1.07 (1.05, 1.09)
rs2487928	KIAA1462	A	G	0.418	4.41E-11	1.06 (1.04, 1.08)
rs2519093	ABO	T	C	0.191	1.19E-11	1.08 (1.06, 1.11)
rs2681472	ATP2B1	G	A	0.201	6.17E-11	1.08 (1.05, 1.10)
rs28451064	KCNE2 (gene desert)	A	G	0.121	1.33E-15	1.14 (1.10, 1.17)
rs2891168	9p21	G	A	0.489	2.29E-98	1.21 (1.19, 1.24)
rs3184504	SH2B3	T	C	0.422	1.03E-09	1.07 (1.04, 1.09)
rs3918226	NOS3	T	C	0.06	1.70E-09	1.14 (1.09-1.19)
rs4252185	PLG	C	T	0.06	1.64E-32	1.34 (1.28, 1.41)
rs4420638	APOE-APOC1	G	A	0.166	7.07E-11	1.10 (1.07, 1.13)
rs4468572	ADAMTS7	C	T	0.586	4.44E-16	1.08 (1.06, 1.10)
rs4593108	EDNRA	C	G	0.795	8.82E-10	1.07 (1.05, 1.10)
rs515135	APOB	C	T	0.79	3.09E-08	1.07 (1.04, 1.10)
rs55730499	SLC22A3-LPAL2-LPA	T	C	0.056	5.39E-39	1.37 (1.31, 1.44)
rs56062135	SMAD3	C	T	0.79	4.50E-09	1.07 (1.05, 1.10)
rs56289821	LDLR	G	A	0.9	4.44E-15	1.14 (1.11, 1.18)
rs56336142	KCNK5	T	C	0.807	1.85E-08	1.07 (1.04, 1.09)
rs663129	PMAIP1-MC4R	A	G	0.26	3.20E-08	1.06 (1.04, 1.08)
rs6689306	IL6R	A	G	0.448	2.60E-09	1.06 (1.04, 1.08)
rs67180937	MIA3	G	T	0.663	1.01E-12	1.08 (1.06, 1.11)
rs6725887	WDR12	C	T	0.11	9.51E-18	1.14 (1.11, 1.18)
rs7212798	BCAS3	C	T	0.15	1.90E-08	1.08 (1.05, 1.11)
rs72689147	GUCY1A3	G	T	0.817	6.07E-09	1.07 (1.05, 1.10)
rs7528419	SORT1	A	G	0.786	1.97E-23	1.12 (1.10, 1.15)

rs7568458	VAMP5-VAMP8-GGCX	A	T	0.449	3.62E-10	1.06 (1.04, 1.08)
rs8042271	MFGE8-ABHD2	G	A	0.9	3.70E-08	1.10 (1.06, 1.14)
rs9349379	PHACTR1	G	A	0.432	1.81E-42	1.14 (1.12, 1.16)
rs9970807	PPAP2B	C	T	0.915	5.00E-14	1.13 (1.10, 1.17)

OR: odds ratio; CI: confidence interval; EAF: effect allele frequency

[a]: Increase in odds of coronary artery disease with increase in per effect allele.

Source of data: Nikpay M, Goel A, Won HH, et al. A comprehensive 1,000 Genomes-based genome-wide association meta-analysis of coronary artery disease. Nat Genet. 2015;47(10):1121-1130.

ESM Table 10 Mendelian randomization estimates of body mass index (SD, 1 SD=4.5 kg/m2) using all 97 BMI-related SNPs from both primary and secondary analyses on cardiovascular risk factors and coronary heart disease

Exposure: BMI (N=339,224)	Odds ratio	95% CI	p-value
**Coronary heart disease (N=60,801 cases and 123,504 controls)**			
Inverse-variance weighted	1.40	1.23, 1.59	<0.001
Weighted median	1.44	1.24, 1.67	<0.001
MR-Egger regression			
slope	1.47	1.21, 1.8	<0.001
Intercept (directional pleiotropy)	-0.002	-0.007, 0.004	0.57
**Type 2 diabetes mellitus (N= 34,840 cases and 114,981 controls)**			
Inverse-variance weighted	1.86	1.35, 2.56	<0.001
Weighted median	2.51	2.09, 3.02	<0.001
MR-Egger regression			
slope	3.17	2.46, 4.09	<0.001
Intercept (directional pleiotropy)	0.98	0.98, 0.99	<0.001
	**Beta**	**95% CI**	**p-value**
**Fasting glucose, mmol/l (N=46,186)**			
Inverse-variance weighted	0.07	0.03, 0.1	<0.001
Weighted median	0.08	0.05, 0.11	<0.001
MR-Egger regression			
slope	0.11	0.05, 0.16	<0.001
intercept	-0.001	-0.003, 0.0003	0.13
**HbA_{1c}, % (N=46,368)**			
Inverse-variance weighted	0.05	0.02, 0.08	0.001
Weighted median	0.09	0.04, 0.14	0.000
MR-Egger regression			
slope	0.08	0.001, 0.15	0.047
intercept	-0.0007	-0.003, 0.001	0.52
**Fasting insulin, log pmol/l (N=46,186)**			
Inverse-variance weighted	0.17	0.13, 0.21	<0.001
Weighted median	0.18	0.12, 0.24	<0.001
MR-Egger regression			
slope	0.17	0.09, 0.25	<0.001
intercept	-0.00002	-0.002, 0.002	0.99
**LDL-C, SD (1 SD= 1.0 mmol/l) (N=188,577)**			
Inverse-variance weighted	-0.047	-0.17, 0.072	0.44
Weighted median	-0.018	-0.078, 0.041	0.55
MR-Egger regression			
slope	-0.100	-0.18, -0.02	0.01
intercept	0.002	-0.001, 0.004	0.16
**HDL-C, SD (1 SD= 0.4 mmol/l) (N=188,577)**			
Inverse-variance weighted	-0.21	-0.29, -0.13	<0.001

Weighted median	-0.21	-0.27, -0.15	<0.001
MR-Egger regression			
slope	-0.22	-0.30, -0.15	<0.001
intercept	0.0005	-0.002, 0.003	0.63
**TG, SD (1 SD= 1.024 mmol/l) (N=188,577)**			
Inverse-variance weighted	0.18	0.12, 0.24	<0.001
Weighted median	0.21	0.16, 0.26	<0.001
MR-Egger regression			
slope	0.18	0.10, 0.25	<0.001
intercept	0.00002	-0.002, 0.002	0.98

SD: standard deviation; CI: confidence interval; BMI: body mass index; LDL-C: low-density lipoprotein cholesterol; HDL-C: high-density lipoprotein cholesterol; TG: triglycerides; HbA_{1c}: glycosylated haemoglobin A_{1c}

ESM Table 11 Multivariate separate-sample Mendelian randomization analysis of the effect of body mass index (per SD, 1 SD=4.5 kg/m2) using all 97 BMI-related SNPs from both primary and secondary analyses on coronary heart disease

	Odds ratio	95% CI	p-value	Mediation effect
MR-IVW regression, crude	1.40	1.23, 1.59	<0.001	
Multivariate model				
(1) adjusted for TG	1.12	0.97, 1.30	0.12	23%
(2) adjusted for HbA_{1c}	1.25	1.10, 1.43	0.001	8%
(3) adjusted for T2DM	1.23	1.07, 1.42	0.004	-
(4) adjusted for TG+HbA_{1c}	1.08	0.93, 1.24	0.30	43%
(5) adjusted for TG+ T2DM	1.09	0.94, 1.27	0.24	-

SD: standard deviation; CI: confidence interval; TG: triglycerides; HbA_{1c}: glycosylated haemoglobin A_{1c}; T2DM: type 2 diabetes mellitus

ESM Table 12. Multivariate separate-sample Mendelian randomization analysis of the effect of body mass index (per SD, 1 SD=4.5 kg/m^2) on coronary heart disease testing potential mediation by LDL-C

	Odds ratio	95% CI	p-value	Mediation effect
**Based on 77 SNPs from the primary analysis**				
MR-IVW regression, crude	1.45	1.27, 1.66	<0.001	
Multivariate model				
adjusted for LDL-C	1.48	1.31, 1.68	<0.001	-6%
**Additionally included 20 SNPs from the secondary analysis**				
MR-IVW regression, crude	1.40	1.23, 1.59	<0.001	
Multivariate model				
adjusted for LDL-C	1.43	1.27, 1.60	<0.001	-3%

SD: standard deviation; CI: confidence interval; LDL-C: low-density lipoprotein cholesterol

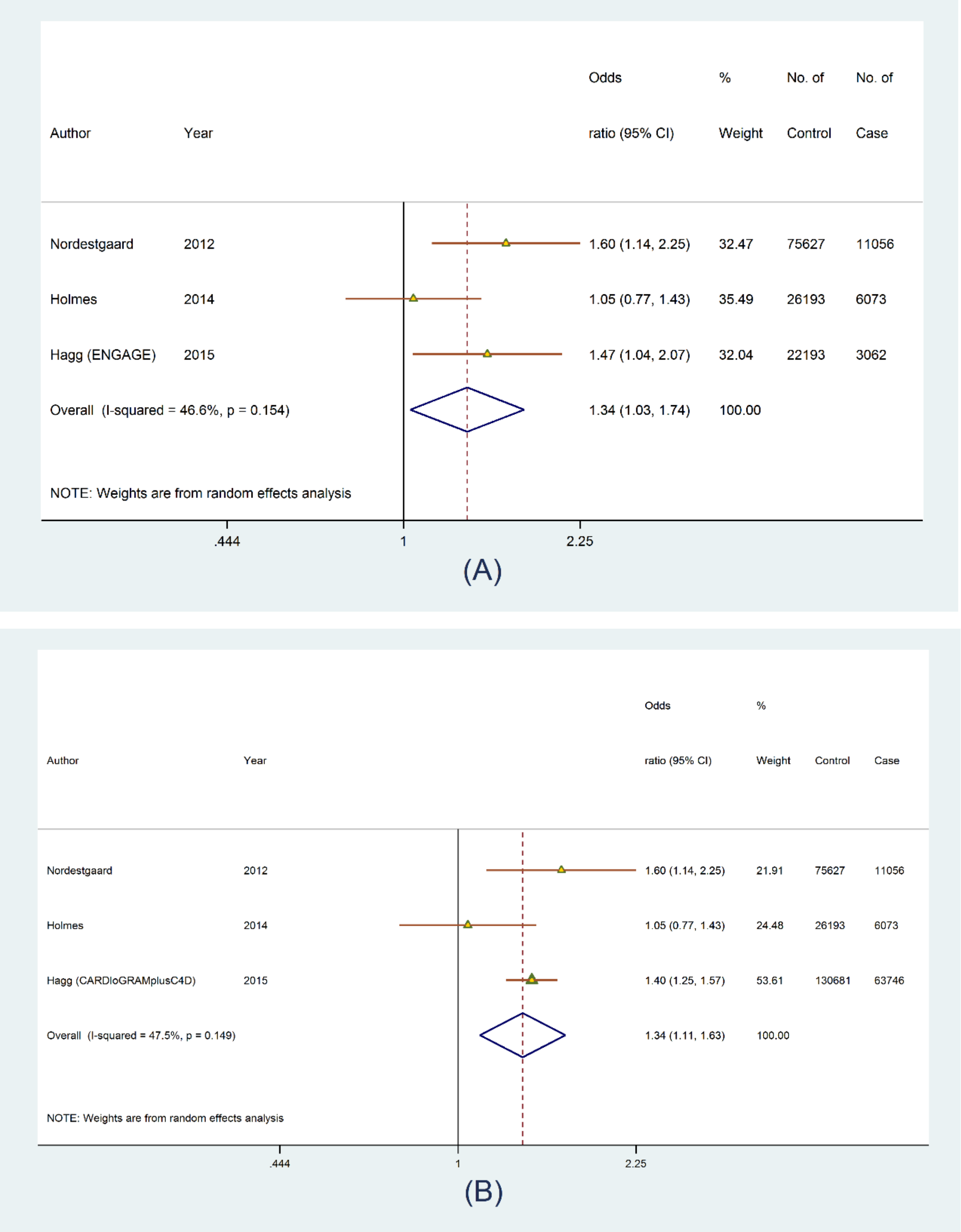

ESM Fig. 1. Meta-analysis of earlier Mendelian randomization studies [4-6] investigating the effect of body mass index (BMI) on coronary heart disease (CHD). Results were represented by odds ratio (OR, 95% confidence interval (CI)) per 4.5 kg/m^2 increase in BMI on the odds of CHD. Results from CARDIoGRAMplusC4D are presented per SD with the SD in the paper given as 4.5. We therefore rescaled results from the other two publications to represent odds

ratios per 4.5 kg/m^2.

Note: Because in Hagg et al. 2015, all genotype consortia data from either ENGAGE or CARDIoGRAMplusC4D and most of the ENGAGE cohorts also contribute to CARDIoGRAMplusC4D, we combined results from the two other studies either with Haag et al. results either from ENGAGE [ESM Fig. 1-(A)] or results from CARDIoGRAMplusC4D [ESM Fig. 1-(B)] separately, so that we were not double counting studies. In all studies the vast majority of participants came from European ancestry individuals,
